# Single-Cell Analyses Reveal Necroptosis's Potential Role in Neuron Degeneration and Show Enhanced Neuron-Immune Cell Interaction in Parkinson's Disease Progression

**DOI:** 10.1155/2023/5057778

**Published:** 2023-12-19

**Authors:** Xiaomei Zeng, Zhifen Han, Kehan Chen, Peng Zeng, Yidan Tang, Lijuan Li

**Affiliations:** ^1^Rehabilitation Medicine Center and Institute of Rehabilitation Medicine, West China Hospital, Sichuan University, Chengdu, China; ^2^Key Laboratory of Rehabilitation Medicine in Sichuan Province, Sichuan University, Chengdu, China; ^3^Department of Ultrasound, Affiliated Hospital of Chengdu University of Traditional Chinese Medicine, Sichuan, Chengdu, China

## Abstract

Parkinson's disease (PD) is a common neuron degenerative disease among the old, characterized by uncontrollable movements and an impaired posture. Although widely investigated on its pathology and treatment, the disease remains incompletely understood. Single-cell RNA sequencing (scRNA-seq) has been applied to the area of PD, providing valuable data for related research. However, few works have taken deeper insights into the causes of neuron death and cell-cell interaction between the cell types in the brain. Our bioinformatics analyses revealed necroptosis-related genes (NRGs) enrichment in neuron degeneration and selecting the cells by NRGs levels showed two subtypes within the main degenerative cell types in the midbrain. NRG-low subtype was largely replaced by NRG-high subtype in the patients, indicating the striking change of cell state related to necroptosis in PD progression. Moreover, we carried out cell-cell interaction analyses between cell types and found that microglia (MG)'s interaction strength with glutamatergic neuron (GLU), GABAergic neuron (GABA), and dopaminergic neuron (DA) was significantly upregulated in PD. Also, MG show much stronger interaction with NRG-high subtypes and a stronger cell killing function in PD samples. Additionally, we identified CLDN11 as a novel interaction pattern specific to necroptosis neurons and MG. We also found LEF1 and TCF4 as key transcriptional regulators in neuron degeneration. These findings suggest that MG were significantly overactivated in PD patients to clear abnormal neurons, especially the NRG-high cells, explaining the neuron inflammation in PD. Our analyses provide insights into the causes of neuron death and inflammation in PD from single-cell resolution, which could be seriously considered in clinical trials.

## 1. Introduction

Parkinson's disease (PD) is the second most reported neurodegenerative disorder, affecting millions of people worldwide every year and is getting more and more prevalent these years [1–4]. It is a neurodegenerative disorder characterized by the death of dopaminergic neurons in the brain, leading to motor and cognitive impairments. The exact cause of the disease is not fully understood, where both genetic and environmental factors are believed to play a role. Based on former research, several cellular pathways have been implicated in the pathogenesis of PD, including oxidative stress, mitochondrial dysfunction, inflammation, and protein misfolding and aggregation. First, oxidative stress has been shown to contribute to dopaminergic neuron death and the accumulation of *α*-synuclein, a protein that is a hallmark of the disease [5]. Second, mitochondrial dysfunction can lead to increased production of ROS as well as impaired energy production and calcium signaling, all of which contribute to cell death [6]. Moreover, many misfolded proteins such as *α*-synuclein are abundant in the brain and comprise the pathological hallmark of PD such as Lewy bodies. The misfolding and aggregation of these misfolded proteins are thought to contribute to the death of dopaminergic neurons in PD [7].

Single-cell RNA sequencing (scRNA-seq) reveals the presence and abundance of RNA at a given moment from a single-cell resolution and has already become a powerful method to understand the pathology of diverse diseases, such as cancers, Alzheimer's disease (AD), and inflammatory bowel disease (IBD) [8–10]. Its high resolution allows for the identification of cell subpopulations and their gene expression signatures, enabling a deeper understanding of cellular heterogeneity and the complex regulatory networks that underlie cellular functions and diseases. One of the main applications of scRNA-seq is in the field of developmental biology, where it has been used to study the differentiation of stem cells into various cell lineages [11]. scRNA-seq has also been used in neurobiology to study the heterogeneity of brain cells and their role in diseases. For example, a study by Lake et al. used scRNA-seq to identify the molecular diversity of different cell types in the brain and to analyze the effects of aging on the gene expression profiles of these cells [12]. scRNA-seq has also been applied in immunology, infectious diseases, and regenerative medicine. As technology continues to improve and become more accessible, it is expected to have a major impact on a wide range of research fields. Among these research studies, many works used scRNA-seq to build an atlas of diseases, producing a lot of data worth further analyzing.

We analyzed published snRNA-seq data from PD patients' samples in this work. 3 groups of degenerative neurons were identified. Differentially expressed genes were analyzed on these cells followed by GO and KEGG enrichment. We found a wide presence of necroptosis in these cells and then carried out cell interaction analysis to study the interactions of MG with these cells. In a word, our work reveals the importance of necroptosis in PD progression from the single-cell aspect and predicts a novel interaction pattern between MG and necroptosis neuron cells.

## 2. Materials and Methods

### 2.1. Data Collection

We acquired midbrain single-nucleus RNA sequencing (snRNA-Seq) data from NCBI (https://www.ncbi.nlm.nih.gov/) using the accession code GSE140231 [13], which include seven health human donors' substantia nigra, GSE157783 [14], which is composed of five PD patients' and six health donors' midbrain, and GSE148434 [15], consisting of six health samples and six PD samples, all of these samples are substantia nigra (Table S1). Because substantia nigra is a part of midbrain, we called our integrated dataset as midbrain dataset. We also defined the healthy or normal sample as term “control,” which means no clinical symptoms related to PD (absence of movement disorder).

### 2.2. Data Preprocessing and Integration

Raw sequencing data were processed using CellRanger-6.1.2. The expression matrix was then imported into R (4.2.1) using Seurat (4.9.9) [16]. We used Seurat package default parameters if no parameters are declared. We removed cells that had fewer than 200 genes or over 4000 genes, as well as those with over 3% of mitochondria genes expressed in them, to ensure that only cells in good condition and not doublets were included. After separately normalizing the data, we merged the Patient and Control expression matrixes together by using Seurat build in function called “FindIntegrationAnchors” with selecting the top 2000 highly variable genes (HVGs). We then scaled the matrix and performed principal component analysis (PCA) using the top 2000 HVGs. We chose the top 15 PCs for downstream cluster identification and visualization. We identified 7 clusters under 0.04 resolution using the “FindClusters” function. All the steps taken after importing the data into R were processed using Seurat internal functions.

### 2.3. Cluster Identification and Gene Ontology Analysis between Clusters

We identified these seven clusters of cells as microglia (MG), oligodendrocyte (OLG), GLU, GABAergic neurons (GABA), dopaminergic neuron (DA), oligodendrocyte precursor cell (OPC) and astrocyte (AST) according to the CellMarker database [17].

Using the Seurat function “FindAllMarkers,” we identified the top 30 markers for each cell type. We then used the clusterProfiler (4.6.2) [18, 19] and genome-wide annotation database to perform GO enrichment analysis between clusters.

### 2.4. Differential Expression Gene (DEG) Analysis in Sub-Clusters between Control and Patient

To analyze a certain cell type, we generated a volcano plot of differentially expressed genes (log2Foldchange >0.58, adjusted *P* Value <0.01) using the Seurat function “FindMarkers.” (Table S2) We then logarithmically transformed the normalized expression matrix by column to make the differences between the two groups clearer in the heatmap. KEGG enrichment analysis was conducted using clusterProfiler [18, 19]. KEGG dotplot and cnetplot were generated using the R package enrichplot. To separate cells in one cluster into two groups with high or low expression of necroptosis-associated genes, we calculated the mean expression level for each gene and assigned cells with more than 60% of genes expressed above the mean as “high” and those with less than 60% as “low.” We then performed PCA of the two groups using the R package “FactoMineR” (2.8) [20] and visualized the results using the R package “factoextra” (1.0.7).

### 2.5. Protein and Protein Interaction Analysis

We found transcription factors which exist in top 200 different expressed genes by using human transcription factors database, humanTFDB [21]. Then, we used STRING [22] database to obtain the protein and protein interaction information with minimum required interaction score >0.4. To make the result clearer and more readable, we used Cytoscape (3.9.1) [23] for further processing. Default mode was used in the analysis.

### 2.6. Cell-Cell Interaction Analysis

We used the R package CellChat (1.6.1) [24] to quantitatively infer and analyze intercellular communication networks from the data. CellChat employs network analysis and pattern recognition approaches to predict major signaling inputs and outputs for cells and how those cells and signals coordinate for functions. CellChat classifies signaling pathways and delineates conserved and context-specific pathways through manifold learning and quantitative contrasts. First, we found identify overexpressed ligands or receptors by using functions called identifyOverExpressedGenes and identifyOverExpressedInteractions. Then, we computed the communication probability via computeCommunProb function. If the interactions appeared in few cells (usually <10), we removed them by filterCommunication function. In the next step, we used computeCommunProbPathway to predict the pathways according to the probable interations. Finally, we calculated the aggregated cell-cell communication network by using aggregateNet function.

## 3. Results

### 3.1. Single-Cell Landscape of Midbrain in PD Patients

**Figure 1:**
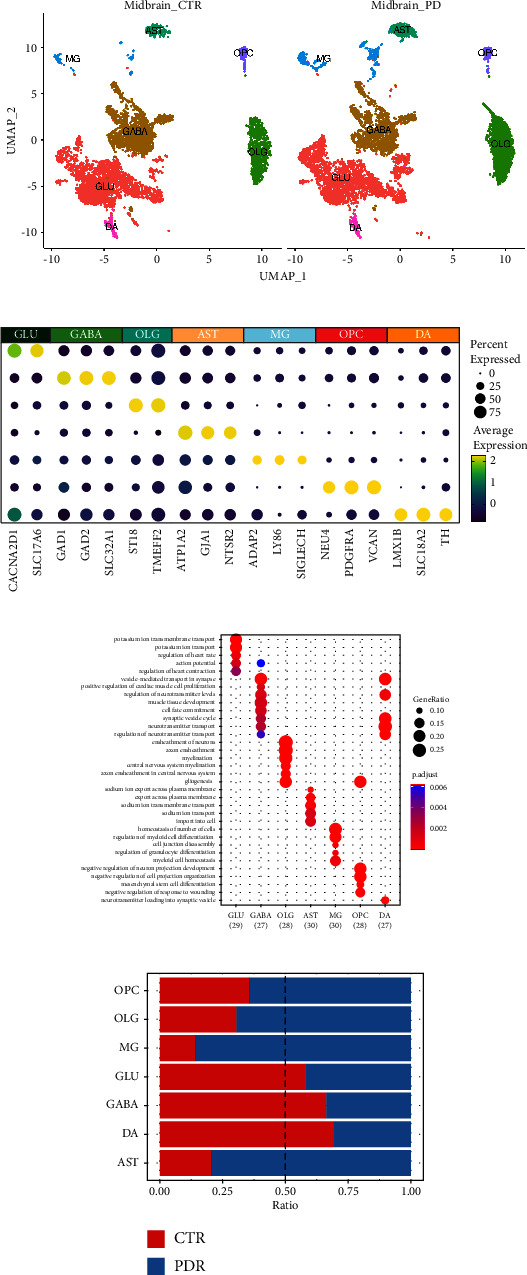
Single cell landscape of midbrain in PD patients. (a) Cell clusters in midbrain between control and PD patients' samples. (b) Cell markers for each cluster. (c) GO enrichment of the clusters. (d) Clusters' proportion changes between PD and control samples. MG ratio increases in PD samples while GLU, GABA, and DA ratios decrease. (CTR: control ratio, PDR: PD ratio).

The main lesions of Parkinson's disease occur in the midbrain. Therefore, in this study, we utilized published single-nucleus sequencing data of midbrain. A total of 14,011 midbrain cells were collected and clustered into seven groups using unsupervised clustering methods. Based on cell markers reported in previous studies, we identified seven distinct cell types, including GABAergic neuron (GABA), glutamatergic neuron (GLU), oligodendrocyte (OLG), oligodendrocyte precursor cell (OPC), astrocyte (AST), microglia (MG), and dopaminergic neuron (DA) (Figure 1(a)). The annotated cell markers are listed in Figure 1(b). We performed GO functional enrichment analysis to identify the main pathways in each of these cell clusters (Figure 1(c)). The enriched pathways corresponded to the known pathways of each cell type, such as myelination of OLG and homeostasis of MG. This demonstrates the accuracy and reliability of our data processing and cluster annotation.

Next, we investigated the changes in cell ratios between PD patient and control samples. Our findings revealed that three major cell types, including GLU, GABA, and DA, were significantly reduced in PD, while MG were the most boosted cells in PD (Figure 1(d)). These intriguing observations piqued our curiosity to study the underlying reasons for these cell proportion changes, and the potential cellular interactions involved.

### 3.2. DEG Analysis and Pathway Enrichment Show Upregulated Necroptosis in GLU Degeneration

**Figure 2:**
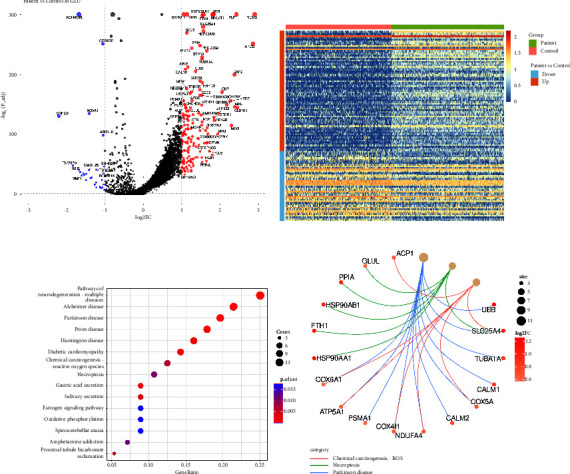
DEG analysis and pathway enrichment of GLU. (a) Volcano plot showing DEGs of GLU between PD and control samples. (b) Heatmap of DEGs in single cells. (c) KEGG enrichment of GLU DEGs. (d) Genes enriched in ROS, necroptosis, and PD pathways.

GLU is the largest cluster in the midbrain and is significantly reduced in PD development. Therefore, we first focused our analysis on the GLU group to understand the mechanisms driving GLU degeneration in PD progression. We analyzed the differentially expressed genes (DEGs) between GLU cells from patient samples and control normal samples (Figure 2(a)). We found that some heat shock proteins, such as HSP90AB1, were significantly upregulated in the PD samples, indicating a higher burden of misfolded abnormal proteins. We also plotted the significant DEGs in single-cell resolution and classified them as upregulated or downregulated genes (Figure 2(b)). To further elucidate the drivers of GLU degeneration, we performed KEGG enrichment analysis for the upregulated genes in PD (Figure 2(c)). Except the reactive oxygen species (ROS) pathway, the necroptosis pathway was also enriched in PD, indicating the necroptosis's role in GLU degeneration. We identified individual genes enriched in the ROS, necroptosis, and PD pathways (Figure 2(d)), and the PD pathway still showed upregulated genes associated with abnormal protein folding.

### 3.3. GLU of Different Necroptosis States Shows Distinct Distribution in PD and Control Samples

**Figure 3:**
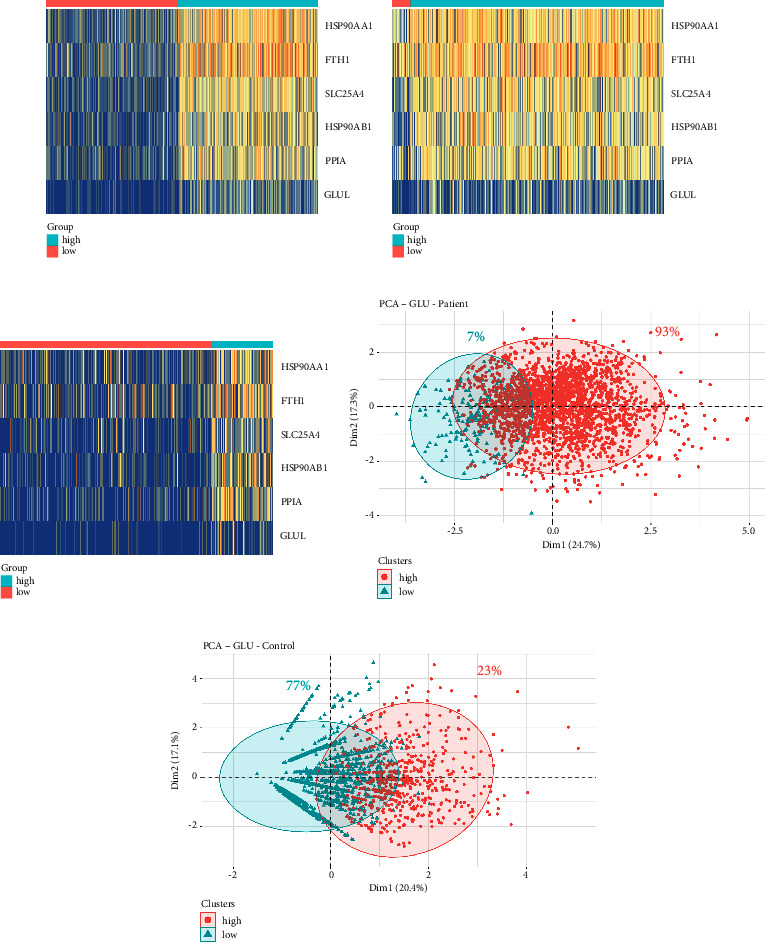
GLU subcluster distribution. (a) NRGs expression between PD and control samples. (b) Heatmap showing two subclusters distribution of GLU in PD samples. (c) Heatmap showing two subclusters of GLU in control samples. (d) PCA of two GLU NRG subclusters in PD samples. (e) PCA of two GLU NRG subclusters in control samples.

In greater detail, we selected the top six enriched genes in the necroptosis pathway and compared their expression levels between patient and control samples (Figure 3(a)). Subsequently, we classified GLU into two subtypes based on their necroptosis state, utilizing the genes we had selected. Cells that exhibited lower expression levels for at least four out of the six genes, relative to the average, were identified as NRG-low subtype, while the remaining cells were identified as NRG-high subtype. The distribution of these subtypes was displayed on a heatmap (Figures 3(b) and 3(c)). We employed PCA to cluster the cells and analyzed the relative proportions of each subtype. Our analysis revealed that NRG-high GLU cells were predominant in the PD brain, whereas NRG-low cells were the primary subtype observed in control samples (Figures 3(d) and 3(e)). These findings suggest that necroptosis may contribute to GLU degeneration.

### 3.4. Necroptosis in DA and GABA Degeneration

**Figure 4:**
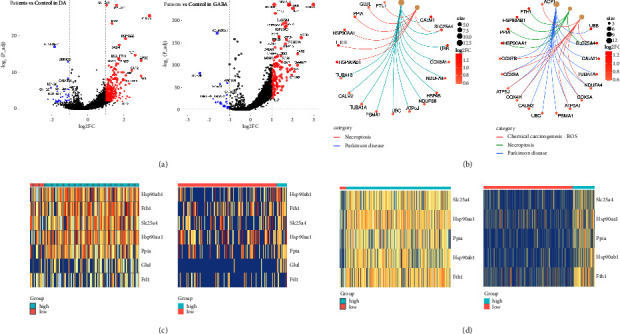
DEG analysis and pathway enrichment of GABA and DA. (a) Volcano plot showing DEGs of DA and GABA between PD and control samples. (b) Heatmap of DEGs enriched in the necroptosis pathway. (c) Heatmap of DA NRG subclusters distribution. (d) Heatmap of GABA NRG subcluster distribution.

In addition to GLU, the other two primary clusters of neurons in the midbrain—dopaminergic neurons (DA) and GABAergic neurons (GABA)—also exhibited a significant reduction in the PD model, which is consistent with the former notion that degeneration of DA neurons is a leading cause of PD [25]. To further investigate changes in these two cell types, we conducted a DEG analysis of DA and GABA (Figures 4(a), S1A). Surprisingly, we also identified necroptosis-related genes, such as HSP90AB1, FTH1, SLC25A4, and PPIA. KEGG analysis also revealed enrichment of the necroptosis pathway (Figures 4(b), S1B). Subsequent classification of DA or GABA neurons based on NRG expression showed a higher proportion of NRG-high cells in the PD group (Figures 4(c) and 4(d), S1C and S1D). These findings suggest that necroptosis is a common occurrence in degenerated neurons during PD progression.

### 3.5. CellChat Analysis Shows Enhanced Cell Interactions between MG and Neuron Cells in PD

**Figure 5:**
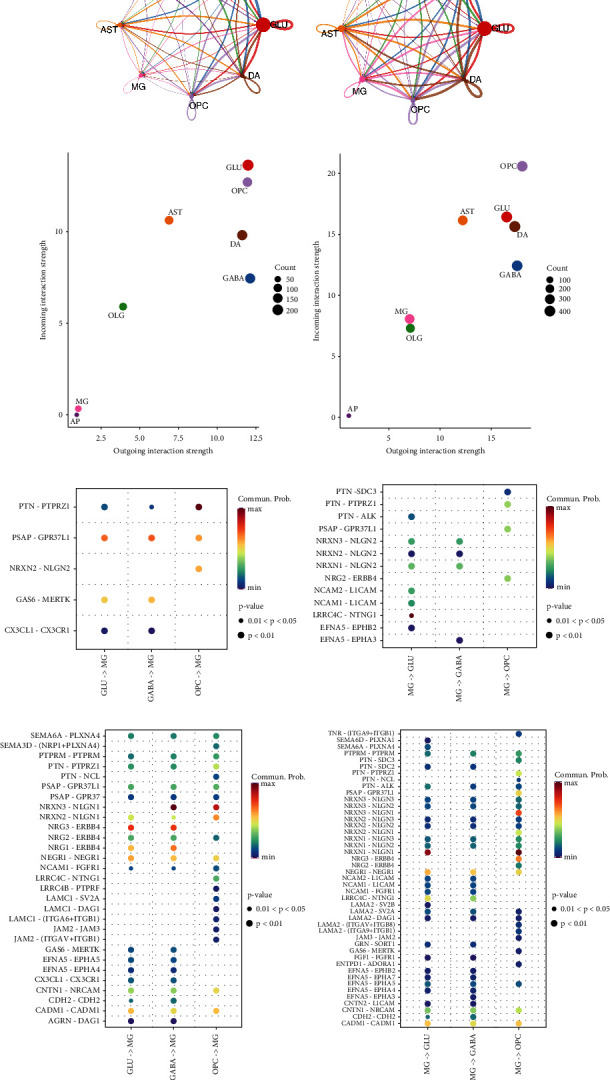
CellChat analysis of clusters within midbrain. (a) Interaction overview between clusters within midbrain. (b) Incoming and outgoing strengths of each cell type. Stronger incoming and outgoing strengths of MG in PD is shown. (c) Interaction pathways between MG and degenerative cells in control samples. (d) Interaction pathways between MG and degenerative cells in PD samples.

Microglia are resident macrophage cells of the brain that serve as the primary immune defense system in the central nervous system. Given the significant number of neuron degeneration observed in PD progression, we postulated that microglia were activated in PD and began attacking neurons, potentially leading to neuron inflammation. To investigate this, we conducted CellChat analysis to study the interactions between neurons and microglia. We discovered that the total interactions were markedly stronger in patient than in control (Figure 5(a)). Additionally, both incoming and outgoing interactions of microglia were higher, indicating an active MG state in PD (Figure 5(b)). We then analyzed the potential molecular interactions between microglia and the three groups of degenerated cells (Figures 5(c) and 5(d)). We detected significantly more possible molecular interactions, indicating enhanced microglia-neuron interactions in PD samples. Among these molecular pathways in PD, CX3CL1 was specifically upregulated in DA, which may play a role in recruiting immune cells. Furthermore, the phagocytosis pathway, such as Gas6-Mertk, was specific to PD-DA with microglia. These results suggest that, in PD progression, DA becomes dysfunctional and releases chemokines, such as CX3CL1, to recruit microglia and is more susceptible to attack by microglia through phagocytosis.

### 3.6. NRG-High Cells Show Stronger Interaction with MG

**Figure 6:**
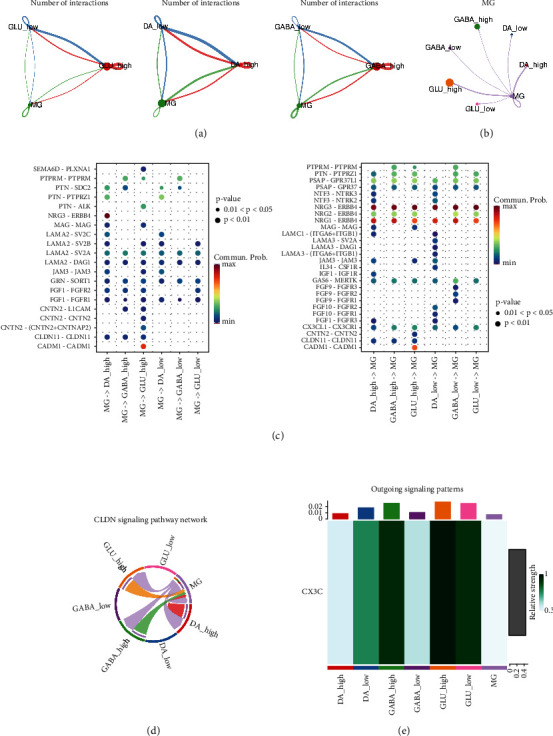
CellChat of NRG subtypes and MG. (a) Total interactions between MG and NRG subtypes. (b) MG outgoing interactions with NRG subtypes showing higher interactions between MG and NRG-high groups. (c) Interaction pathways between MG and NRG subtypes. (d) CLDN pathway between MG and NRG subtypes. (e) CX3C pathway of subtypes.

To further address the necroptosis's effect on neuron inflammation, we examined the interactions between NRG-high neurons and MG. NRG-high cells exhibited a significantly greater number of total cell interactions with MG (Figure 6(a)). MG's outgoing interactions with NRG-high cells were particularly higher compared to NRG-low cells (Figure 6(b)). Detailed interaction pathways were analyzed and the larger number of molecular interactions was identified between MG and NRG-high cells (Figure 6(c)). In addition, we discovered a potential pathway, CLDN11, which was specifically associated with NRG-high cells and microglia (Figure 6(d)). This gene is not well-studied and may play an important role in the enhanced MG-necroptosis cells interactions in the PD pathological process. Furthermore, the CX3C pathway was also stronger between NRG-high cells and MG (Figure 6(e)), indicating that cells undergoing necroptosis have a greater ability to attract microglia. Notably, DA did not show the same pattern here, which may result from the small cell number in the NRG-low DA. Collectively, these findings showed robust necroptosis cell-MG interactions in PD tissues.

### 3.7. Analysis of Transcription Factors Driving the Necroptosis in Degenerated Neurons

**Figure 7:**
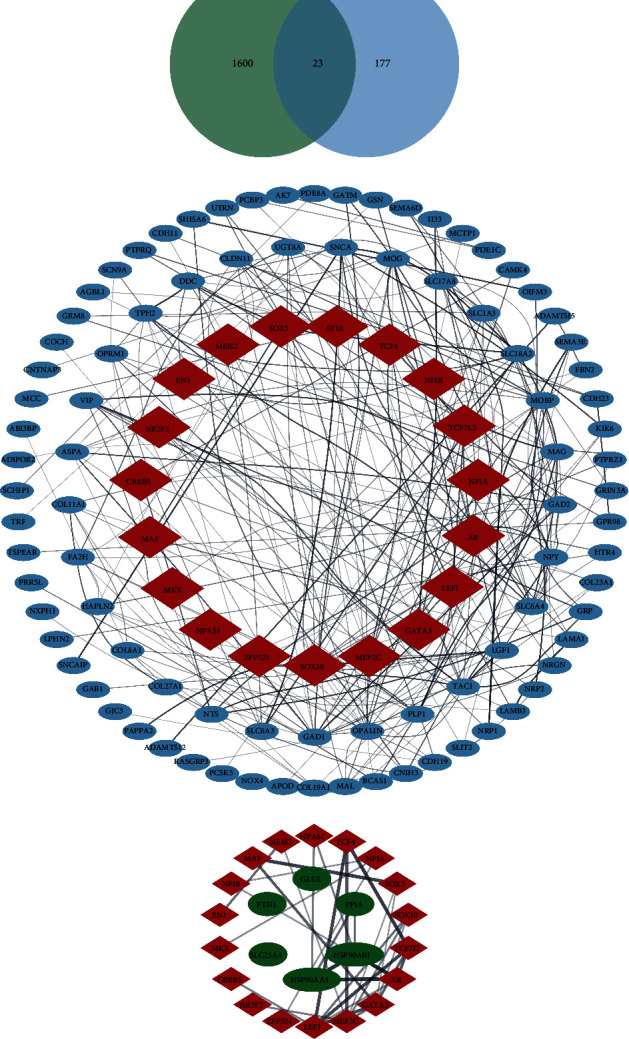
Analysis of hub transcription factors driving the necroptosis in degenerated neurons. (a) Regulated transcription factors were obtained from DEGs; 23 TFs were identified. (b) PPI network of the TFs and DEGs in degenerated neurons. NRGs were centered. (c) PPI network of the NRGs and their related TFs in degenerated neurons. TFs were centered.

We then conducted an analysis of the transcriptional regulation in the PD samples to elucidate the mechanisms of neuron necroptosis. Differentially expressed genes (DEGs) and transcription factors (TFs) were compared to identify the distinctly expressed transcription factors (Figure 7(a)). Additionally, we performed a protein-protein interaction analysis to examine the downstream genes regulated by these TFs (Figure 7(b)). Our findings indicated the involvement of 23 central TFs in the degeneration process in PD, controlling approximately 100 downstream genes. Furthermore, most of the enriched NRGs were regulated by these TFs, with LEF1, TCF4, and GATA3 playing significant roles in the regulatory network (Figure 7(c)).

### 3.8. Microglia Functions Were Enhanced in PD Patients

**Figure 8:**
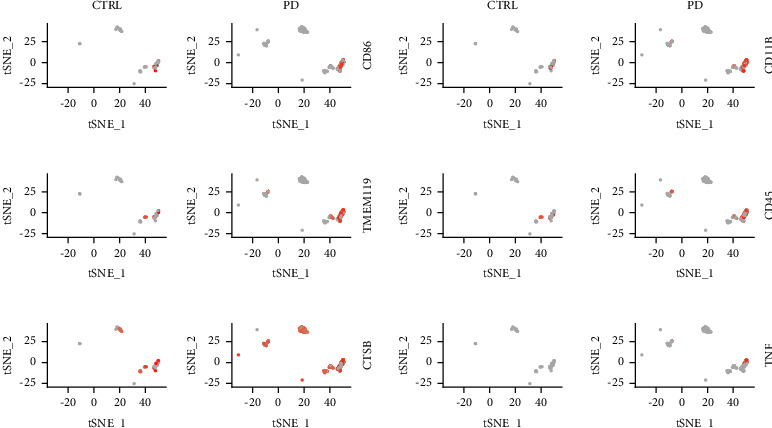
PD patients have stronger microglia function. (a–f). Activation markers such as CD86 and effector markers such as CTSB of microglia were upregulated in PD patients.

Upon activation, microglia upregulate CD86, TMEM119, CD11B, and CD45, and they exert their cytotoxic effects on target cells through the release of various substances, including TNF-*α*, glutamate, cathepsin B, superoxide, or nitric oxide (Figure 8) [26, 27]. To further validate the microglia's excessive activation in the progression of Parkinson's disease (PD), we investigated key activation markers and functional indicators relevant to cell killing at the single-cell level. All markers exhibited significant upregulation in PD patients compared to control samples, suggesting a heightened state of microglial activation and increased cell-killing activity within the PD brain.

## 4. Discussion

Necroptosis is a type of programmed cell death that has gained significant attention in recent years due to its distinct molecular mechanisms and implications in various physiological and pathological processes. Unlike apoptosis, which is a well-known and regulated form of cell death characterized by controlled dismantling of the cell, necroptosis is an inflammatory and lytic form of cell death. It represents an alternative cell death pathway that can be activated when cells are unable to undergo apoptosis, often triggered by various stress signals, such as infection, inflammation, or cellular damage [28–30].

Necroptosis has been implicated in various pathological conditions, including ischemic injuries, viral infections, and inflammatory disorders. Its dysregulation has been linked to the pathogenesis of conditions such as Alzheimer's disease, and inflammatory bowel diseases. Consequently, understanding the molecular mechanisms underlying necroptosis may hold therapeutic potential for developing novel treatments to modulate cell death and inflammation in these disorders [31]. However, it is not widely investigated how necroptosis will influence Parkinson's disease.

Some clinical studies have observed significant increases in necroptosis-related genes (NRGs) in postmortem examinations of Parkinson's disease (PD) patients, such as MLKL [25]. However, our understanding of the expression profiles of these genes across different cell types within the brain and how their expressions can influence neighboring cells remains limited. Although a recent study reported the enrichment of NRGs in PD samples using bulk RNA-seq data, it lacked deeper insights into cell interaction changes and robust verification at the single-cell level [32].

In our study, we comprehensively examined the expression patterns of NRGs among midbrain neurons in PD samples from a single-cell perspective and identified some NRGs upregulated in PD patients. Among these genes, GLUL encodes glutamate-ammonia ligase which is important in RIPK3-mediated metabolic enzyme regulation [33]. It helps the production of reactive oxygen species (ROS) and enhanced necroptosis [34]. HSP90 increases MLKL oligomerization and plasma membrane translocation to trigger necroptosis and is required for TNF-induced necroptosis [35, 36]. FTH1 and FTL1 encode the heavy and light chain of ferritin, respectively. The acccumulation of ferritin can elicit oxidative inflammation and NF-*κ*B-TNF*α* pathway, which may further trigger necroptosis [37]. PPIA (cyclophilin A) acts as an immune inflammatory mediator that secretes proinflammatory cytokines induced by oxidative stress [38–41]. SLC25A4 encodes the adenine nucleotide translocator-1 (ANT1), which is involved in metabolism via the regulation of ATP/ADP release from mitochondria and in regulated cell death as part of the mitochondrial permeability transition pore [42]. We further used these genes to study the cell subtypes distribution within the brain and found NRG-low subtype's replacement by NRG-high subtypes. Our cell-cell interaction analyses showed more activated microglia and more interaction of microglia with the degenerative neurons. These findings shed light on the significant role that necroptosis may play in neuron degeneration and microglia (MG)-mediated immune clearance. Additionally, we identified novel cell interaction pathways and central transcription factors in PD that warrant further investigation.

However, our analysis has potential limitations due to the limited sample number and heterogeneity between samples. Also, the interaction predicted by CellChat may not be the same as the physiological condition. More experimental verification could be carried out regarding the enhanced cell interactions. Future research should focus on understanding the mechanisms underlying the widespread occurrence of necroptosis in PD and explore the potential application of necroptosis inhibitor drugs as a therapeutic approach. Moreover, investigating the mechanistic role of the CLDN pathway and upregulated transcription factors in PD development could provide valuable insights for disease pathogenesis and treatment strategies.

## 5. Conclusion

We analyzed published data on PD patients and found widespread necroptosis genes upregulated in three main degenerative neuron types in the midbrain. Our cell-cell interaction analysis showed that MG, the immune cells of the brain, have much stronger interactions with the neurons undergoing necroptosis. All these results implied that the main neurons in the midbrain turn to a dysfunctional state through a necroptosis-related mechanism, which activates MG to clear the dysfunctional neurons in PD, leading to neuron degeneration and inflammation. We also identified CLDN11 as a potential interaction pathway specific between MG and NRG-high cells. It may play a role in mediating the enhanced cell interactions in PD. In a word, our results suggest that the necroptosis cell-microglia axis may play a crucial role in PD progression. Our results showed broad application prospects in drug development targeting necroptosis [33].

## Data Availability

The data supporting the findings of this study can be found in the NCBI (https://www.ncbi.nlm.nih.gov/) of GEO with accession code GSE157783, GSE140231 and GSE148434.

## Additional Points

*Internet Resources*. [1] Carlson M (2019). org.Mm.eg.db: Genome wide annotation for Mouse. R package version 3.8.2. https://bioconductor.org/packages/org.Mm.eg.db/. [2] Yu G (2023). enrichplot: Visualization of Functional Enrichment Result. R package version 1.20.0, https://yulab-smu.top/biomedical-knowledge-mining-book/. [3] pheatmap. R package version 1.0.12 https://cran.r-project.org/web/packages/pheatmap/index.html. [4] factoextra: Extract and visualize the results of multivariate data analyses. R package version 1.0.7 https://cran.r-project.org/web/packages/factoextra/index.html

## Supplementary Materials

Supplementary Materials Figure S1: DA and GABA subclusters distribution. A: Heatmap showing DEGs expression of DA and GABA in single cells. B: KEGG enrichment of DEGs. C: PCA of two DA subclusters between PD and control samples. D: PCA of two GABA subclusters between PD and normal samples. Table S1: Detailed information of the patient samples and control samples were listed. Dataset column shows the sources of the samples. The sample name column shows the name of each sample appeared in the dataset. The brain position where samples came from includes Midbrain and substantia nigra. The patients and control samples' gender and ages were also provided. Table S2: Differential expression genes between Control and Parkinson's disease patients in cell subtypes (GABA, DA and GLU respectively) with statistical test.
