# Supplementary figures and images for: Single-Cell Analyses Reveal Necroptosis's Potential Role in Neuron Degeneration and Show Enhanced Neuron-Immune Cell Interaction in Parkinson's Disease Progression
Source: Parkinsons Dis. 2023 Dec 19;2023:5057778. doi: 10.1155/2023/5057778 (PMC10751163; doi:10.1155/2023/5057778)

**A**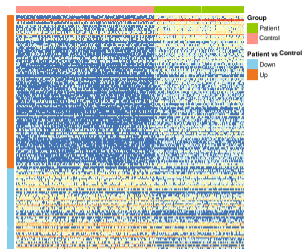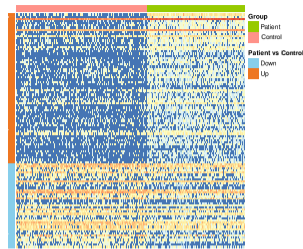**B**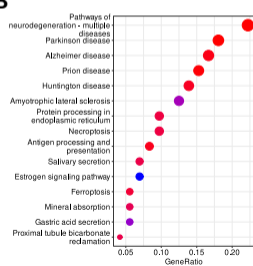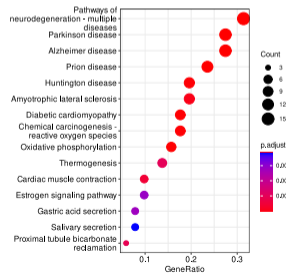**C**

PCA - DA - Patient

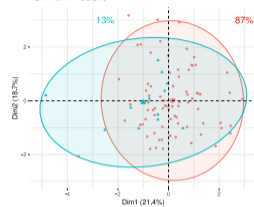

PCA - DA - Control

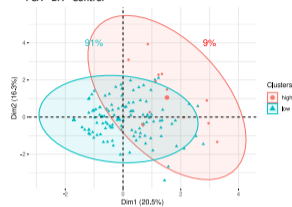**D**

PCA - GABA - Patient

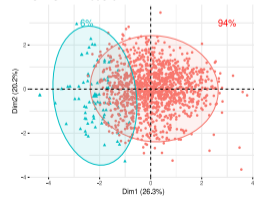

PCA - GABA - Control

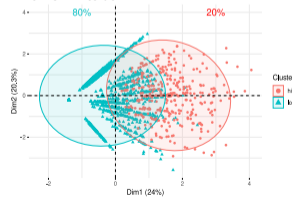

Supplement: Supplementary Materials — Figure S1: DA and GABA subclusters distribution. A: Heatmap showing DEGs expression of DA and GABA in single cells. B: KEGG enrichment of DEGs. C: PCA of two DA subclusters between PD and control samples. D: PCA of two GABA subclusters between PD and normal samples. Table S1: Detailed information of the patient samples and control samples were listed. Dataset column shows the sources of the samples. The sample name column shows the name of each sample appeared in the dataset. The brain position where samples came from includes Midbrain and substantia nigra. The patients and control samples' gender and ages were also provided. Table S2: Differential expression genes between Control and Parkinson's disease patients in cell subtypes (GABA, DA and GLU respectively) with statistical test. [file 5057778.f1.zip › Fig-S1.pdf]
